# Protecting Animals by Vaccination

**Published:** 1882-07

**Authors:** 


### EDITORIAL DEPARTMEMT.

### PROTECTING ANIMALS BY VACCINATION.

PASTEUR'S method of vaccinating against splenic fever or anthrax was described in our last issue. Since that time it has been tried quite extensively not only in France under Pasteur's direction, but in Hungary and in Prussia.

In the latter country the results were as follows: Fifty sheep and twelve cows were taken, and divided into two equal lots. The first lot of twenty-five sheep and six cows were vaccinated with the cultivated virus of anthrax prepared by Pasteur. Two weeks later they were vaccinated again. For Pasteur has found that a double or "secondary" vaccination is necessary to make the protection more sure. Three sheep died after the second vaccination.

Three weeks later all the forty-seven sheep and twelve cows were inoculated with virulent anthrax blood. Of the protected animals, none died. Of the non-protected, three cows and all the sheep died.

This can be best shown by a table:

| Animals experimented on: 12 cows, 50 sheep. | | Inocuulated with anthrax blood. | Death resulted in |
|---|---|---|---|
| Vaccinated twice: 6 cows, 25 sheep. | Died from 2d vaccination: 3 sheep. | 6 cows. | o |
| | | 22 sheep. | o |
| Unvaccinated: 6 cows, | | 6 cows. | 3 cows. |
| 25 sheep. | | 25 sheep. | 25 sheep. |

In Hungary, similar results were obtained. The conclusions are that the protective vaccination has a value; but when a farmer has his herds thus treated, he must expect to *lose ten per cent.* by the measure. In some countries anthrax destroys sixty per cent. of the herds. Hence vaccination is the better of the two evils there.

